# Perceived stress, unhealthy eating behaviors, and severe obesity in low-income women

**DOI:** 10.1186/s12937-015-0110-4

**Published:** 2015-12-03

**Authors:** Andrea S. Richardson, Joanne E. Arsenault, Sheryl C. Cates, Mary K. Muth

**Affiliations:** RAND Health, RAND Corporation, 570 Fifth Ave. #600, Pittsburgh, PA 15213 USA; U.C. Davis, Program in International and Community Nutrition, 3217A Meyer Hall One Shields Ave., Davis, CA 95616 USA; RTI International, Food and Nutrition Policy Research Program, 3040 E. Cornwallis Rd., Research Triangle, Park, NC 27709-3910 USA

**Keywords:** Stress, Obesity, Severe obesity, Eating behaviors, Diet, WIC

## Abstract

**Background:**

Stress has been associated with poor eating behaviors and diet quality, as well as high body mass index (BMI). Low-income women may be particularly vulnerable to stress and severe obesity. Yet it is unknown how stress increases the risk of severe obesity through disordered eating behaviors and poor diet quality or through mechanisms independent of diet.

**Methods:**

We examined cross-sectional data from women (*n* = 101) with a child enrolled in the Special Supplemental Nutrition Program for Women, Infants, and Children in Cumberland County, North Carolina (spring 2012). We collected measured heights and weights to calculate BMI. Using structural equation modeling, we differentiated pathways from stress to weight status: (1) indirectly through eating behaviors (cognitive restraint, emotional eating, and uncontrolled eating) and diet quality, which we examined with the Healthy Eating Index 2010 and 24-h dietary recalls, and (2) directly through possible unmeasured risk factors independent of diet. The analysis controlled for race/ethnicity, income, age, whether the dietary recall day was typical, and whether the respondent completed one or two 24-h dietary recalls.

**Results:**

Perceived stress was positively associated with uncontrolled eating (β = 0.38, *p* < 0.001) and emotional eating (β = 0.50, *p* < 0.001). However, higher stress was not associated with weight status through eating behaviors and diet quality. Independent of eating behaviors and diet quality, stress was positively associated with severe obesity (β = 0.26, *p* = 0.007).

**Conclusions:**

Improving stress coping strategies for low-income women may improve eating behaviors and reduce severe obesity.

**Electronic supplementary material:**

The online version of this article (doi:10.1186/s12937-015-0110-4) contains supplementary material, which is available to authorized users.

## Background

Obesity prevalence remains high; approximately 35 % of American adults 20 years of age or older are obese [1]. Severe obesity prevalence, defined by a body mass index (BMI) of 40 kg/m^2^ or more, is rising faster than moderate obesity [2–7] and is expected to increase 130 % in the adult U.S. population by 2030 [8]. Severely obese individuals experience serious health complications such as diabetes, hypertension, hyperlipidemia, asthma, arthritis [9], and even reductions in life expectancy [10, 11]. Stress has been associated with weight gain [12] and with potentially obesogenic eating behaviors, including higher energy intake [13], increased saturated fat and sugar intake [14], and poor diet quality [15, 16]. However, stress increases physiologic responses that are independent of eating behaviors and diet. For example, cortisol increases lipogenesis [17, 18]. Thus, stress might influence severe obesity through the following pathways: (1) through eating behaviors and diet quality and (2) through biological processes. Yet few studies have been able to disentangle how stress may be associated with severe obesity through eating behaviors and diet quality from other stress-related risk factors that are independent of diet. Low-socioeconomic women are a particularly vulnerable population that is also disproportionately burdened by severe obesity [19]. Given the difficulty of treating severe obesity [20, 21], as well as its serious cardiometabolic [9, 11] and psychological comorbidities [22–24], it is critical to identify risk factors that may contribute to severe obesity.

A pathway from stress to severe obesity may operate through eating behaviors [25] if people turn to less healthy and obesogenic dietary behaviors as a means of coping with stress. High perceived stress (upper quartile), versus normal stress, has been associated with worse diet quality [26], greater intake of snack foods and lower intake of fruit [27, 28], increased disinhibition [29], and binge eating [30]. The association between stress and increased food intake was once thought to occur through impaired cognitive restraint; however, the evidence supporting that association has recently been questioned [31]. Instead, stress may activate reward signal pathways in the brain that increase intake of highly palatable foods [32], high-fat foods [33], and snack foods and decrease intake of fruit [28].

Three eating behavior constructs are restraint, disinhibition, and hunger, which can be assessed by a psychometric questionnaire [34]. Restraint is the conscious restriction of food intake to control body weight. Disinhibition is the tendency to overeat in response to different stimuli, such as when an individual is presented with palatable foods or is under emotional distress. Hunger is the susceptibility to eat in response to perceived physiological symptoms signaling the need for food. Of the three constructs, disinhibition has been associated with excess body weight [35, 36]. In particular, disinhibition strongly predicted weight gain and current BMI in adult women [35].

However, stress could increase severe obesity risk through other pathways not related to diet. For example, physiologic stress responses increase activation of the central sympathetic nervous system and the hypothalamic-pituitary-adrenal (HPA) axis, which can increase cortisol secretion that then is followed by visceral fat accumulation [18].

Low-income women are disproportionately burdened by severe obesity and are faced with multiple stressors that may be due to financial constraints and lack of power. Low-income mothers of young children may be particularly stressed if faced with being the sole provider for their children [37]. How stress might increase severe obesity through eating behaviors and diet quality while accounting for unmeasured pathways (e.g., biological) in low-income women is poorly understood.

To address this gap, we used structural equation modeling (SEM) to simultaneously test multiple pathways from stress to severe obesity. Specifically, in addition to testing the indirect pathway from perceived stress to severe obesity through eating behaviors and diet quality, we tested the direct pathway from perceived stress to severe obesity to capture non-diet-related risk factors that are unmeasured in this study. We hypothesized that high levels of perceived stress would be associated with severe obesity both indirectly, through eating behaviors and diet quality, and directly, through non-diet-related risk factors, in low-income women. In addition, we hypothesized that the mechanisms may operate differently for moderate and severe obesity.

## Methods

### Study population

The study population was a convenience sample of adult (18 years or older) women who had a child up to age 5 enrolled in the Special Supplemental Nutrition Program for Women, Infants and Children (WIC) in Cumberland County, NC. WIC staff at the clinics helped identify women eligible for the study who were scheduled for a WIC clinic visit on the days when study staff would be present and asked eligible women if they would be interested in participating in the study. Information on the number of women who were asked to participate in the study but declined was not recorded, so we are unable to calculate a response rate. Study staff enrolled 101 women from March through May 2012. We included women who had a child 6 months of age or older enrolled in WIC who were not pregnant and not breastfeeding, because these factors may affect eating behaviors. Subjects received $25 for completing the in-person interview and dietary recall. The study was approved by RTI International’s Institutional Review Board.

### Anthropometry

WIC clinic staff measured height to 0.1 in. (0.254 cm) using a stadiometer (Perspective Enterprises) and weight to 0.1 lb (0.45 kg) using a digital scale (Healthometer). We defined weight status with the following categories:

- underweight/normal weight: BMI ≤ 24 kg/m^2^,
- overweight: BMI ≥ 25 and < 30 kg/m^2^,
- moderately obese: BMI ≥ 30 and < 40 kg/m^2^, and
- severely obese: BMI ≥ 40 kg/m^2^.

### Assessments and questionnaires

#### Demographics

We administered a questionnaire that included questions about age, race/ethnicity, educational level, and annual household income (Additional file 1). Participants were asked how old they were based on the following categories (18–24, 25–29, 30–34, 35–44 years). We also collected the following additional demographics: race/ethnicity (non-Hispanic white, non-Hispanic black, non-Hispanic other, Hispanic), educational level (no college vs. some college or more), and annual household income (≤ $9,999, $10,000–$39,999, ≥ $40,000). We created a variable to flag those with (*n* = 59, 58 %) and without (*n* = 42, 42 %) two 24-h dietary recalls.

### Perceived stress questions

The questionnaire included the 14-item Perceived Stress Scale, a validated measure of the degree to which situations are appraised as stressful [38]. Responses were scored on a 5-point scale from “never” to “very often” on questions such as “In the last month, how often have you felt that you were unable to control the important things in your life?” Some questions received reverse scoring with a high score for “never,” such as “In the last month, how often have you felt confident in your ability to handle your personal problems?” Scores were totaled, and the scale ranges from 0 to 56. The Cronbach alpha internal consistency coefficient of the scale was 0.84, which is above the acceptable cutoff of 0.70.

### Eating behaviors questions

The questionnaire included the 18-item Three-Factor Eating Questionnaire [39], an abbreviated version of the original 51-item questionnaire [34] that measures three eating behaviors: emotional eating, uncontrolled eating, and cognitive restraint. Emotional eating is measured by three questions (e.g., “When I feel blue, I often overeat”). Uncontrolled eating is measured by nine questions (e.g., “Sometimes when I start eating, I just can’t seem to stop”). Cognitive restraint is measured by six questions (e.g., “I do not eat some foods because they make me fat”). We scored item responses from 1 to 4 based on degree of agreement with the statement or frequency of the feeling or behavior. For each factor, we summed scores and transformed raw scales to a scale of 0 to 100. Therefore, high scores reflect more of the eating behavior. The Cronbach alpha internal consistency coefficients of the scales were 0.83 for emotional eating, 0.77 for uncontrolled eating, and 0.75 for cognitive restraint.

### Dietary recall

We collected a 24-h dietary recall at the time of the interview in the WIC clinic using the United States Department of Agriculture’s (USDA’s) Automated Multiple Pass Method software [40]. Interviewers used the USDA food model booklet and measuring cups and spoons to aid the women in estimating portion sizes. We converted food intakes to nutrient and food group intakes using USDA’s SurveyNet software. The software provides coding for approximately half of foods, and the remaining foods were manually coded and checked by a dietitian on the study team. In addition, the program flags high consumption amounts, and these were verified with the interviewers. Respondents reported whether the day of the recall was typical. If the respondent agreed to a second interview, we contacted them by phone a week later for a second 24-h dietary recall.

We calculated the Healthy Eating Index-2010 (HEI-2010) [41] using the 24-h dietary recall data. The HEI-2010 consists of 12 components that are scored such that higher scores correspond to better adherence to the 2010 Dietary Guidelines for Americans [42]. Each component has a score from either 0 to 5 (servings of total fruit, whole fruit, total vegetables, dark green and orange vegetables and legumes, total grains, whole grains), 0 to 10 (servings of milk, meat and beans, oils, saturated fat, and sodium), or 0 to 20 (calories from solid fats, alcoholic beverages, and added sugars [SoFAAS]). The sum of the 12 component scores is the total HEI-2010 score, ranging from 0 to 100. Higher component scores for HEI moderator components, such as SoFAAS, represent lower intake of these components and better adherence to dietary recommendations. For the respondents with two 24-h dietary recalls, we calculated the average of the two HEI-2010 scores.

### Statistical analysis

To describe our study population, we calculated means and standard deviations (continuous variables) and percentages (categorical variables) of all variables included in our model using Stata 13.0 (StataCorp, College Station, TX).

SEM is a pathway-based approach that can handle multiequation models and allows estimation among latent (unobserved) and observed variables of multiple effects transmitted over combinations of paths [43]. We used Mplus version 7.11 [44] with maximum likelihood and missing values; we set statistical significance at *p* < 0.05 (2-sided).

**Fig. 1:**
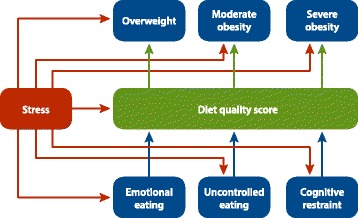
Conceptual model of pathways from stress to weight status^1^. ^1^We controlled for the following confounders along the perceived stress to weight status pathways: income, age, race/ethnicity, whether the dietary recall day was a typical day and if the respondents had one or two 24-hour dietary recalls, marital status, and income

We used SEM to examine pathways from perceived stress to weight status, including direct and indirect pathways through eating behaviors and diet quality. Figure 1 presents our conceptual model of hypothesized direct and indirect pathways. We included in our model the three constructs of eating behaviors (emotional, uncontrolled, and cognitive restraint). Although these eating behaviors are salient to our research question, we acknowledge that they do not represent the full universe of eating behaviors. For example, stress-induced food cravings could affect diet quality without necessarily being one of the three behaviors we modeled. Therefore, we included the direct pathway from stress to diet quality to capture pathways from stress to obesity through unmeasured dietary behaviors that we cannot model with these data.

We addressed confounding of associations between perceived stress and weight status by controlling for income, age, race/ethnicity, marital status, whether the dietary recall day was a typical day, and if the respondents had one or two 24-h dietary recalls. In a preliminary estimation of the model, education did not change the associations between perceived stress and weight status, so we did not include it in the final model. We defined good model fit as chi-square test *X*^*2*^ > 0.05, root Mean Square Error of Approximation (RMSEA) < 0.06 [45], and Comparative Fit Index (CFI) [46] and Tucker-Lewis Index (TLI) [47] values approaching 1.0.

## Results

### Descriptive statistics

Overall, our study population was 67 % nonwhite or Hispanic (Table 1). Most respondents were under 30 years of age, did not have a college education, were married, and had a household annual income less than $40,000. On average, the participants’ BMI was 32 kg/m^2^. About 40 % reported that the day of their 24-h dietary recall was not a usual day of dietary intake. All scales ranged from 0 to 100, and on average, respondents scored 26 on perceived stress (higher is worse), 44 on cognitive restraint (higher is better), 31 on uncontrolled eating (higher is worse), 37 on emotional eating (higher is worse), and 44 on the HEI-2010 (higher is better).

**Table 1:** Study population characteristics

	Total^a^	Under/Normal weight^b^	Overweight^c^	Moderately obese^d^	Severely obese^e^	*p*-value^f^
*N*	101	17	22	27	15	
Race/ethnicity—*N* (%)						0.39
Non-Hispanic white	33 (32.7)	8 (47.1)	6 (27.3)	13 (27.7)	6 (40.0)	
Non-Hispanic black	46 (45.5)	4 (23.5)	9 (40.9)	25 (53.2)	8 (53.3)	
Non-Hispanic other	6 (5.9)	1 (5.9)	3 (13.6)	2 (4.3)	0 (0.0)	
Hispanic	16 (15.8)	4 (23.5)	4 (18.2)	7 (14.9)	1 (6.7)	
Age (years)—*N* (%)						0.09
18–24	35 (34.7)	8 (47.1)	12 (54.5)	11 (23.4)	4 (26.7)	
25–29	37 (36.6)	5 (29.4)	8 (36.4)	20 (42.6)	4 (26.7)	
30–34	15 (14.9)	2 (11.8)	1 (4.5)	10 (21.3)	2 (13.3)	
35–44	14 (13.9)	2 (11.8)	1 (4.5)	6 (12.8)	5 (33.3)	
Some college or more—*N* (%)	47 (46.5)	9 (52.9)	6 (27.3)	24 (51.1)	8 (53.3)	0.24
Married—*N* (%)	62 (61.4)	14 (82.4)	15 (68.2)	26 (55.3)	7 (46.7)	0.13
Income—*N* (%)						0.70
Less than $10,000	31 (32.3)	4 (23.5)	4 (20.0)	17 (37.8)	6 (42.9)	
$10,000 to $39,999	50 (52.1)	10 (58.8)	12 (60.0)	21 (46.7)	7 (50.0)	
$40,000 or more	15 (15.6)	3 (17.6)	4 (20.0)	7 (15.6)	1 (7.1)	
Reported unusual diet intake—*N* (%)	37 (36.6)	8 (47.1)	7 (31.8)	17 (36.2)	5 (33.3)	0.78
Perceived stress^g^—mean (SD)	26.3 (0.8)	25.9 (2.2)	24.0 (1.6)	26.0 (1.1)	31.2 (2.2)	0.07
Cognitive restraint^h^—mean (SD)	44.0 (2.1)	39.9 (5.7)	44.2 (4.8)	42.9 (2.7)	51.9 (6.6)	0.43
Uncontrolled eating^h^—mean (SD)	30.6 (1.6)	39.9 (3.6)	25.9 (2.8)	29.0 (2.7)	31.9 (3.6)	0.05
Emotional eating^h^—mean (SD)	36.9 (3.1)	26.1 (5.4)	29.3 (6.6)	40.4 (4.8)	48.9 (7.1)	0.10
Healthy Eating Index^i^—mean (SD)	43.9 (1.6)	44.4 (4.9)	43.6 (3.4)	41.8 (1.8)	50.2 (5.1)	0.37

^a^Mean BMI = 32 kg/m^2^

^b^BMI ≤ 24 kg/m^2^

^c^Overweight: BMI ≥ 25 and < 30 kg/m^2^

^d^Moderately obese: BMI ≥ 30 and < 40 kg/m^2^

^e^Severely obese: BMI ≥ 40 kg/m^2^

^f^Comparisons across weight status: Chi-square for categorical variables, multivariate analysis of variance for continuous variables

^g^The 14-item Perceived Stress Scale, a validated measure of the degree to which situations are appraised as stressful [38]

^h^The 18-item Three-Factor Eating Questionnaire [39], an abbreviated version of the original 51-item questionnaire [34]

^i^The Healthy Eating Index-2010 (HEI-2010) [41], an average of two scores used for 59 women (58 % of our total sample) who completed two 24-h dietary recalls

Few women were under- or normal weight (17 %); most were either overweight (22 %) or moderately obese (27 %), and 15 % were severely obese. Severely obese women were mostly non-Hispanic white and non-Hispanic black. Although differences were not statistically significant across weight status, moderately and severely obese women were slightly older and less likely to have an income of $40,000 or more compared with respondents who were not obese. Overweight women were least likely to be college educated compared with other respondents. Under- and normal weight women were more likely to be married and report a nontypical day compared with other respondents. Severely obese women scored highest on perceived stress, cognitive restraint, uncontrolled eating, emotional eating, and the HEI-2010. Both under/normal weight and overweight women scored the lowest on perceived stress, cognitive restraint, uncontrolled eating, and emotional eating. However, the moderately obese women scored the lowest on the HEI-2010.

### Structural equation modeling

**Fig. 2:**
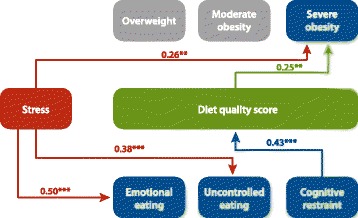
Structural equation modeling results of multiple pathways from stress to weight status^1^. Note: Statistically nonsignicant associations are not shown. ^1^We controlled for the following confounders along the perceived stress to weight status pathways: income, age, race/ethnicity, whether the dietary recall day was a typical day and if the respondents had one or two 24-hour dietary recalls, marital status, and income

Model fit for the SEM presented in Fig. 2 was good (*X*^*2*^*p* = 0.17, RMSEA = 0.04, CFI = 0.93, TLI = 0.89). Perceived stress was directly and positively associated with severe obesity (β = 0.26, *p* = 0.007), emotional eating (β = 0.50, *p* < 0.001), and uncontrolled eating (β = 0.38, *p* < 0.001). Counter to our hypothesis, we did not observe an indirect pathway from perceived stress to weight status through eating behaviors and diet quality. Higher cognitive restraint was associated with higher diet quality (β = 0.43, *p* < 0.001). However, women with a higher diet quality score were more likely to be severely obese (β = 0.25, *p* = 0.007) than women with a lower diet quality score. We present a summary of all parameter estimates in Table 2.

**Table 2:** Standardized estimates from structural equation models examining the pathways from perceived stress to weight status through eating behaviors and diet quality

Dependent variables	Explanatory variables	β	S.E.	*p*-value
Weight status (Normal/underweight is referent)				
Overweight	Perceived stress	−0.06	0.10	0.54
	Diet quality score	−0.08	0.10	0.08
	Income			
	Less than $10,000	−0.19	0.11	0.08
	$10,000 to $39,999 (referent)	0.00	—	—
	$40,000 or more	0.03	0.10	0.77
	Race/Ethnicity			
	Non-Hispanic white (referent)	0.00	—	—
	Non-Hispanic black	0.06	0.10	0.52
	Non-Hispanic other	0.09	0.10	0.34
	Hispanic	0.01	0.10	0.95
	Age (years)			
	18–24	0.14	0.10	0.18
	25–29 (referent)	0.00	—	—
	30–34	−0.14	0.10	0.16
	35–44	−0.14	0.10	0.17
	Two versus one 24-h dietary recall	−0.10	0.09	0.27
Moderate obesity	Perceived stress	−0.15	0.10	0.14
	Diet quality score	−0.15	0.10	0.15
	Income			
	Less than $10,000	0.16	0.11	0.14
	$10,000 to $39,999 (referent)	0.00	—	—
	$40,000 or more	0.07	0.10	0.51
	Race/Ethnicity			
	Non-Hispanic white (referent)	0.00	—	—
	Non-Hispanic black	0.10	0.10	0.30
	Non-Hispanic other	0.12	0.10	0.23
	Hispanic	−0.02	0.10	0.85
	Age (years)			
	**18–24**	**−0.22**	**0.10**	**0.03**
	25–29 (referent)	0.00	—	—
	30–34	0.13	0.10	0.21
	35–44	−0.03	0.10	0.79
	Two versus One 24-h Dietary Recall	0.11	0.09	0.25
Severe obesity	**Perceived stress**	**0.26**	**0.10**	**0.007**
	**Diet quality score**	**0.25**	**0.09**	**0.007**
	Income			
	Less than $10,000	0.09	0.11	0.39
	$10,000 to $39,999 (referent)	0.00	—	—
	$40,000 or more	−0.09	0.10	0.36
	Race/Ethnicity			
	Non-Hispanic white (referent)	0.00	—	—
	Non-Hispanic black	−0.03	0.10	0.76
	Non-Hispanic other	−0.13	0.90	0.17
	Hispanic	−0.07	0.09	0.47
	Age (years)			
	18–24	0.02	0.10	0.86
	25–29 (referent)	0.00	—	—
	30–34	−0.02	0.10	0.81
	**35–44**	**0.20**	**0.10**	**0.04**
	Two versus one 24-h dietary recall	0.00	0.09	0.97
Perceived stress	Income			
	**Less than $10,000**	**0.28**	**0.10**	**0.005**
	$10,000 to $39,999 (referent)	0.00	—	—
	$40,000 or more	0.06	0.10	0.56
	Race/Ethnicity			
	Non-Hispanic white (referent)	0.00	—	—
	Non-Hispanic black	−0.05	0.10	0.65
	Non-Hispanic other	−0.04	0.10	0.71
	Hispanic	−0.01	0.10	0.94
	Age (years)			
	18–24	−0.10	0.10	0.32
	25–29 (referent)	0.00	—	—
	30–34	0.15	0.10	0.16
	35–44	0.18	0.10	0.08
	Dietary recall day was a typical day	−0.05	0.10	0.61
	Two versus one 24-h dietary recall	0.04	0.39	0.07
Cognitive restraint	Perceived stress	−0.12	0.10	0.25
Uncontrolled eating	**Perceived stress**	**0.38**	**0.09**	**<.001**
Emotional eating	**Perceived stress**	**0.50**	**0.08**	**<.001**
Diet quality score	Perceived stress	−0.18	0.10	0.08
	**Cognitive restraint**	**0.43**	**0.08**	**<.001**
	Uncontrolled eating	−0.05	0.10	0.58
	Emotional eating	0.01	0.10	0.89
	Dietary recall day was a typical day	0.06	0.09	0.53
	Two versus one 24-h dietary recall	0.03	0.09	0.74

Bold text indicates statistical significance at the 5 % level

## Discussion

In a sample of low-income women with children, we found that perceived stress was directly and positively associated with severe obesity, independent of eating behaviors and diet quality. In addition, perceived stress was directly and positively associated with unhealthy eating behaviors. However, we found no evidence that perceived stress influenced weight status through eating behaviors and diet quality. This suggests that non-diet-related behaviors (e.g., physical activity) or physiologic mechanisms (e.g., cortisol) associated with high levels of perceived stress may also contribute to severe obesity. Thus, improving stress coping strategies to help low-income women feel less stressed may improve stress-induced nondietary behaviors and physiologic responses that underlie severe obesity.

Approximately 40 % of women in the study were either moderately or severely obese, and 15 % were severely obese, almost double the national average among women 20 years or older, 8.3 % [48]. However, women in our sample are socioeconomically disadvantaged, and our results are similar to a previous national estimate that 42 % of women living in low-income households are obese [1]. Although there is no recommended overall criterion to indicate adherence to Dietary Guideline using the HEI score, the mean HEI-2010 score of 44 among women in this study was lower than the mean score of 56 for all adults from the National Health and Nutrition Examination Survey 2003–2004 [37]. However, low-income adults have been shown to have lower HEI scores than adults with high income [37].

Our findings support previous findings that women who are stressed are more likely to have emotional and uncontrolled eating behaviors. In their review, Greeno and Wing [25] concluded that stress leads to overeating. Perceived stress was found to be associated with higher frequency of binge eating in a study with 62 obese, mostly white women aged 20 to 64 years [30]; with emotional eating in a study with 159 African American adults from the Washington, DC, area [13]; and with emotional eating, independent of BMI, in a study with 517 minority students in Los Angeles [49]. Yet the mechanisms by which stress influences eating behaviors are not fully understood. Some possible scenarios are that perceived stress can increase greater cortisol reactivity [50] susceptibility to negative moods and self-medication [51], increased disinhibition [52, 53], and increased food cravings [54]. We did not observe indirect pathways from stress to severe obesity through eating behaviors, nor was higher stress associated with lower diet quality. We also did not expect that higher diet quality would be associated with severe obesity. However, 9 of the 12 HEI-2010 components indicate higher consumption of certain foods, and all but one of the components are scaled by caloric intake. Thus, it could be that a person could consume more foods and energy than they need and still score high on the HEI-2010. Alternatively, severely obese individuals may underreport their intake in dietary recalls [40].

Our findings highlight the fact that the effect of perceived stress on dietary behaviors and obesity is complex. Although stress can induce unhealthy eating behaviors, high levels of stress may also affect nondietary factors that increase the likelihood of being severely obese. Thus, it is important to understand and disentangle stress-induced dietary versus nondietary mechanisms to effectively tailor stress coping strategies to reduce severe obesity. The women in our study have children enrolled in WIC, through which they receive food vouchers, nutrition and health education, and health referrals. Integrating stress coping counseling into the WIC services could complement nutrition education. However, WIC would need increased financial resources to hire trained staff and to expand their services, but the multiple benefits to participants’ mental, physical, and eating behaviors could outweigh the costs.

Healthy eating and dietary behaviors are crucial for optimal health and not just to maintain a healthy weight. For example, individuals with poor dietary behaviors can maintain a healthy weight yet suffer from other cardiometabolic disorders (e.g., hypertension) [55]. Further, children often learn eating and dietary behaviors from their mothers [56]. Thus, it is critical to support low-income mothers’ healthy eating and dietary behaviors so they can model healthy behaviors for their children.

Although eating behaviors contribute to overall diet quality, the research examining the association between stress and dietary quality in women is limited. Some studies have examined stress and diet quality in adolescents [15] and in high-income working adults [16], but findings are inconsistent. Mixed findings may relate to how stress and depression might interact with diet quality and BMI in different socioeconomic subgroups [26, 57]. Socioeconomically disadvantaged populations experience more stress and might be more depressed than advantaged populations. A meta-analysis found that low-socioeconomic-status individuals had an increased likelihood of being depressed [58]. Further, a study of low-income Supplemental Nutrition Assistance Program participants living in Pittsburgh found that depression was a strong predictor of poor diet quality and high BMI [59]. This study is one of few that examined both dietary behaviors and BMI outcomes; however, the study did not test whether depression is associated with higher BMI through lower diet quality.

Here, we evaluated a pathway integrating perceived stress with eating behaviors, diet quality, and weight status. We simultaneously tested associations 1) between perceived stress and severe obesity through dietary behaviors (unhealthy eating behaviors and diet quality) and 2) between perceived stress and severe obesity. We observed a direct and positive association between high levels of perceived stress and severe obesity, independent of the associations through unhealthy eating behaviors. Our study is cross-sectional, so we may miss longtime dietary behaviors that contribute to weight gain. Thus, the direct association between perceived stress and severe obesity may reflect confounding by unmeasured longtime obesogenic dietary behaviors. In contrast, other non-diet-related factors may be associated with high levels of stress and severe obesity. For example, low-income women with high levels of stress may not have time for physical activity. In this case, the direct association between high levels of perceived stress and severe obesity may reflect confounding associations with a lack of physical activity that we were not able to include in our model. Low-income women who are stressed because of a lack of financial resources may also lack physical activity time, which would increase obesity risk. Beyond behaviors, physiologic mechanisms could play a role, such as stress-induced cortisol secretion, which increases lipogenesis [18], thereby increasing the likelihood of being severely obese.

We observed associations for eating behaviors, diet quality, and severe obesity but not for eating behaviors, diet quality, and overweight or moderate obesity. More women in our sample were overweight and moderately obese than severely obesity, so it is unlikely that the lack of associations is due to low sample size and power. The difference in associations suggests that different mechanisms may be operating between stress and moderate obesity than between stress and severe obesity.

Our study has some limitations. In addition to the cross-sectional design, what we found in our convenience sample may not generalize to all low-income mothers or women. Also, we did not have 2 days of dietary recall from our whole sample, and 1 day of recall may not reflect typical diet quality. Diet quality indices are global measures of diet; however, people’s diets can vary greatly day to day [60]. However, among the 59 respondents who completed both days of 24-h dietary recall, the HEI-2010 score from the interview day was not statistically significantly different from the HEI-2010 score calculated from the 24-h dietary recall collected 1 week later (analysis-of-variance test *p* = 0.29). In addition, we controlled for whether the woman reported that the recall took place on a typical day, and because the first recall was collected in person and the second was collected by phone, we controlled for whether the woman completed one or two 24-h dietary recalls.

Our sample was relatively small, which prohibited subgroup analyses by race/ethnicity that could underlie different dietary behaviors in response to stress. Race/ethnicity could play a role in perceived weight and behaviors to control weight [61]. Findings from a recent study in a large nationally representative sample of adults suggest that among obese adults, blacks intended to lose weight less frequently than whites [61]. Race/ethnicity also moderated associations between obesity and depression [62] and between the additive effects of anxiety and depression on BMI [63]. As perceived stress relates to mental health and as intention to lose weight relates to dietary behaviors, race/ethnicity could also be an important contextual determinant of how women’s perceived stress relates to BMI through dietary behaviors. This research question warrants attention in future studies with larger sample sizes.

We lacked information on physical activity, which is important to energy balance and weight status. However, people who are physically active also tend to have healthy diet behaviors and weight status, so we may have captured some of the respondents’ propensity to be physically active with their eating behaviors and diet quality. The small sample size limits subgroup analyses such as analysis by age groups or comparison of under- versus normal weight. Only three participants who were classified as under/normal weight were underweight, so it is unlikely they are confounding the estimates. Indeed, when we ran the model excluding the underweight women, the estimates did not change (data not shown). Further, our limited sample size may have affected our ability to detect statistically significant indirect associations from perceived stress to weight status through eating behaviors and diet quality. Nevertheless, we found that stress negatively affects diet behaviors in low-income mothers of young children and increases their likelihood of being severely obese.

## Conclusion

Our results show that low-income mothers are disproportionately burdened with stress and severe obesity. Behavioral interventions aimed at reducing stress may improve dietary behaviors and reduce severe obesity in low-income mothers.

## Additional file

Additional file 1:
**Eating habits study questionnaire.** (DOC 101 kb)

## Abbreviations

BMI: body mass index
CFI: comparative fit index
HEI: healthy eating index
HPA: hypothalamic-pituitary-adrenal
RMSEA: root mean square error of approximation
SEM: structural equation modeling
SoFAAS: solid fats, alcoholic beverages, and added sugars
USDA: United States Department of Agriculture
WIC: special supplemental nutrition program for women, infants and children
