# Supplementary material for: Perceived stress, unhealthy eating behaviors, and severe obesity in low-income women
Source: Nutr J. 2015 Dec 3;14:122. doi: 10.1186/s12937-015-0110-4 (PMC4668704; doi:10.1186/s12937-015-0110-4)
Supplement: Additional file 1: — Eating habits study questionnaire. (DOC 101 kb) [file 12937_2015_110_MOESM1_ESM.doc]

**Eating Habits Study**

Date: ____________________

Interview Start Time: ____________

Interview End Time: _____________

**RTI International**

**Re-Screening Questions**

1. Are you 18 or older?

Yes

No 🡪 **[Terminate]**

2. Are you currently pregnant?

Yes 🡪 **[Terminate]**

No

3. Have you given birth in the past 6 months?

Yes 🡪 **[Terminate]**

No

4. Are you currently breastfeeding?

Yes 🡪 **[Terminate]**

No

5. Do any of the children in your household receive WIC benefits?

Yes

No 🡪 **[Terminate]**

| This survey asks about your eating habits and coping skills and some general questions about you and your household. The survey will take about 15-20 minutes to fill out. We will then ask you about the foods and drinks you ate over the last 24 hours.  Remember, all of your answers will be kept private and your responses will not affect any program benefits that you receive. You may skip any questions you do not want to answer.  For the first set of questions, I am going to read a statement. For each statement, please tell me whether you think the statement is definitely true, mostly true, mostly false, or definitely false. Select the best response for each question. |
| --- |

E1. What is your favorite food? _____________. When I smell [FAVORITE FOOD], I find it very difficult to keep from eating, even if I have just finished a meal.

| 1. Definitely true | 2. Mostly true | 3. Mostly false | 4. Definitely false |
| --- | --- | --- | --- |

E2. I take small helpings on purpose as a means of controlling my weight.

| 1. Definitely true | 2. Mostly true | 3. Mostly false | 4. Definitely false |
| --- | --- | --- | --- |

E3. When I feel anxious or nervous, I find myself eating.

| 1. Definitely true | 2. Mostly true | 3. Mostly false | 4. Definitely false |
| --- | --- | --- | --- |

E4. Sometimes when I start eating, I just can’t seem to stop.

| 1. Definitely true | 2. Mostly true | 3. Mostly false | 4. Definitely false |
| --- | --- | --- | --- |

E5. Being with someone who is eating often makes me hungry enough to eat also.

| 1. Definitely true | 2. Mostly true | 3. Mostly false | 4. Definitely false |
| --- | --- | --- | --- |

E6. When I feel sad or down, I often overeat.

| 1. Definitely true | 2. Mostly true | 3. Mostly false | 4. Definitely false |
| --- | --- | --- | --- |

E7. What is a food that you really like but don’t eat often because it costs a lot? ___________. When I see [THIS FOOD], I often get so hungry that I have to eat right away.

| 1. Definitely true | 2. Mostly true | 3. Mostly false | 4. Definitely false |
| --- | --- | --- | --- |

E8. I get so hungry that my stomach often feels like a bottomless pit.

| 1. Definitely true | 2. Mostly true | 3. Mostly false | 4. Definitely false |
| --- | --- | --- | --- |

E9. I am always hungry so it is hard for me to stop eating before I finish the food on my plate.

| 1. Definitely true | 2. Mostly true | 3. Mostly false | 4. Definitely false |
| --- | --- | --- | --- |

E10. When I feel lonely, I make myself feel better by eating.

| 1. Definitely true | 2. Mostly true | 3. Mostly false | 4. Definitely false |
| --- | --- | --- | --- |

E11. I hold back at meals on purpose in order not to gain weight.

| 1. Definitely true | 2. Mostly true | 3. Mostly false | 4. Definitely false |
| --- | --- | --- | --- |

E12. I do not eat some foods because they make me fat.

| 1. Definitely true | 2. Mostly true | 3. Mostly false | 4. Definitely false |
| --- | --- | --- | --- |

E13. I am always hungry enough to eat at any time.

| 1. Definitely true | 2. Mostly true | 3. Mostly false | 4. Definitely false |
| --- | --- | --- | --- |

E14. How often do you feel hungry? Would you say . . .

| 1. Only at meal times | 2. Sometimes between meals | 3. Often between meals | 4. Almost always |
| --- | --- | --- | --- |

E15. How often do you avoid “stocking up” on tempting foods? By stocking up, I mean buying a lot of something to have on hand.

| 1. Almost never | 2. Seldom | 3. Usually | 4. Almost always |
| --- | --- | --- | --- |

E16. How likely are you to eat less than you want on purpose? Would you say . . .

| 1. Unlikely | 2. Slightly likely | 3. Moderately likely | 4. Very likely |
| --- | --- | --- | --- |

E17. Do you binge, even though you are not hungry? By binge, I mean eating a lot without any control.

| 1. Never | 2. Rarely | 3. Sometimes | 4. At least once a week |
| --- | --- | --- | --- |

E18. On a scale of 1 to 8, where 1 means no control in eating, that is, eating whatever you want, whenever you want it, and 8 means total control, that is, always limiting what you eat and never “giving in,” what number would you give yourself?

| 1 | 2 | 3 | 4 | 5 | 6 | 7 | 8 |
| --- | --- | --- | --- | --- | --- | --- | --- |
| No control in eating |  |  |  |  |  |  | Total  control |

| The next set of questions ask you about your feelings and thoughts during the past month. For each question, tell me how often you felt or thought a certain way using the following responses: never, almost never, sometimes, fairly often, or very often. Treat each as a separate question and give your best estimate of how many times you felt a certain way. |
| --- |

S1. In the last month, how often have you been upset because of something that happened unexpectedly?

| 1. Never | 2. Almost never | 3. Sometimes | 4. Fairly often | 5. Very often |
| --- | --- | --- | --- | --- |

S2. In the last month, how often have you felt that you were unable to control the important things in your life?

| 1. Never | 2. Almost never | 3. Sometimes | 4. Fairly often | 5. Very often |
| --- | --- | --- | --- | --- |

S3. In the last month, how often have you felt nervous and “stressed”?

| 1. Never | 2. Almost never | 3. Sometimes | 4. Fairly often | 5. Very often |
| --- | --- | --- | --- | --- |

S4. In the last month, how often have you dealt successfully with irritating life hassles?

| 1. Never | 2. Almost never | 3. Sometimes | 4. Fairly often | 5. Very often |
| --- | --- | --- | --- | --- |

S5. In the last month, how often have you felt that you were effectively dealing with important changes that were taking place in your life?

| 1. Never | 2. Almost never | 3. Sometimes | 4. Fairly often | 5. Very often |
| --- | --- | --- | --- | --- |

S6. In the last month, how often have you felt confident in your ability to handle your personal problems?

| 1. Never | 2. Almost never | 3. Sometimes | 4. Fairly often | 5. Very often |
| --- | --- | --- | --- | --- |

S7. In the last month, how often have you felt that things were going your way?

| 1. Never | 2. Almost never | 3. Sometimes | 4. Fairly often | 5. Very often |
| --- | --- | --- | --- | --- |

S8. In the last month, how often have you found that you could not deal with all of the things that you had to do?

| 1. Never | 2. Almost never | 3. Sometimes | 4. Fairly often | 5. Very often |
| --- | --- | --- | --- | --- |

S9. In the last month, how often have you been able to control irritations in your life?

| 1. Never | 2. Almost never | 3. Sometimes | 4. Fairly often | 5. Very often |
| --- | --- | --- | --- | --- |

S10. In the last month, how often have you felt that you were on top of things?

| 1. Never | 2. Almost never | 3. Sometimes | 4. Fairly often | 5. Very often |
| --- | --- | --- | --- | --- |

S11. In the last month, how often have you been angered because of things that happened that were outside of your control?

| 1. Never | 2. Almost never | 3. Sometimes | 4. Fairly often | 5. Very often |
| --- | --- | --- | --- | --- |

S12. In the last month, how often have you found yourself thinking about things that you have to accomplish?

| 1. Never | 2. Almost never | 3. Sometimes | 4. Fairly often | 5. Very often |
| --- | --- | --- | --- | --- |

S13. In the last month, how often have you been able to control the way you spend your time?

| 1. Never | 2. Almost never | 3. Sometimes | 4. Fairly often | 5. Very often |
| --- | --- | --- | --- | --- |

S14. In the last month, how often have you felt difficulties were piling up so high that you could not overcome them?

| 1. Never | 2. Almost never | 3. Sometimes | 4. Fairly often | 5. Very often |
| --- | --- | --- | --- | --- |

| The next set of questions asks about you and your household. Remember, the answers to all the questions will be kept private. |
| --- |

D1. I’m going to read a list of age categories. Please stop me when I get to your age.

1. 18–24

2. 25–29

3. 30–34

4. 35–39

5. 40–44

6. 45–49

7. 50–54

8. 55–59

9. 60–64

10. 65–69

11. 70–74

12. 75+

D2. Are you Hispanic or Latino?

1. Hispanic or Latino

2. Not Hispanic or Latino

D3. What is your race? You can select one or more.

1. American Indian or Alaska Native

2. Asian

3. Black or African American

4. Native Hawaiian or other Pacific Islander

5. White or Caucasian

D4. Were you born in the United States?

1. Yes

2. No

D5. Which of the following best describes where you live?

1. Civilian housing

2. Military housing, on base

3. Military housing, off base

D6. What is the highest grade or level of school you have completed or the highest degree you have received?

1. Less than 9th grade

2. 9–11th grade

3. High school graduate or GED equivalent

4. Some college or associates degree

5. College graduate or above

D7. Which of the following best describes your marital status now?

1. Married

2. Widowed

3. Divorced

4. Separated

5. Never married

6. Living with significant other

D8. In the past month, how many people have lived in your household more than half of the time? When counting the number of adults, do **NOT** include your spouse or significant other if deployed.

Number of adults, 18+ yrs _____

Number of children, < 5 yrs _____

Number of children, 5–17 yrs _____

D9. What was your household’s total income last year before taxes? Please include all types of income received by household members, including all earnings, pensions, child support, and cash welfare benefits such as TANF and SSI. Do **NOT** include the value of SNAP, WIC, Medicaid, or public housing. Please stop me when I reach your household’s total income for last year. Was it . . .

1. Less than $10,000

2. $10,000 to $19,999

3. $20,000 to $29,999

4. $30,000 to $39,999

5. $40,000 to $49,999

6. $50,000 to $59,999

7. $60,000 to $69,999

8. $70,000 to $79,999

9. $80,000 or more

D10. Do you or any member of your household currently receive Food Stamp, SNAP, or EBT benefits?

1. Yes

2. No

D11. Are you currently dieting to lose weight?

1. Yes

2. No

M1. Are you currently serving in the military?

1. Yes

2. No 🡪 **GO TO QUESTION M8**

M2. Are you currently:

1. On active duty and not a member of the National Guard or Reserve

2. A member of the National Guard or Reserve in a full-time active duty program, that is, Active Guard Reserve, Full-Time Support, or Active Reserve

3. A traditional National Guard or Reserve member; for example, drilling unit, Individual Mobilization Augmentation, or Individual Ready Reserve

M3. During your active duty career, were you ever deployed?

1. Yes

2. No 🡪 **GO TO QUESTION M8**

M4. Were you deployed for more than 30 days in a row?

1. Yes, in past two years

2. Yes, but it was more than two years ago

3. No 🡪 **GO TO QUESTION M6**

M5. How many times were you deployed for more than 30 days in a row? ____

M6. Were you ever deployed to a combat zone?

1. No🡪 **GO TO QUESTION M8**

2. Yes, deployed to Iraq and/or Afghanistan

3. Yes, deployed to a combat zone other than Iraq or Afghanistan

M7. During your active duty career, how many times were you deployed to a combat zone?

_________

**If respondent answered QD7 married, separated or live-in significant other, continue, otherwise, end survey portion of interview.**

M8. Is your spouse or significant other currently serving in the military?

1. Yes

2. No **🡪 GO TO QUESTION DS1**

M9. Is your spouse or significant other currently:

1. On active duty and not a member of the National Guard or Reserve

2. A member of the National Guard or Reserve in a full-time active duty program, that is, Active Guard Reserve, Full-Time Support, or Active Reserve

3. A traditional National Guard or Reserve member; for example, drilling unit, Individual Mobilization Augmentation, or Individual Ready Reserve

M10. During your spouse or significant other’s active duty career, has she/he ever been deployed?

1. Yes

2. No 🡪 **GO TO QUESTION DS1**

M11. Has your spouse or significant other been deployed for more than 30 days in a row?

1. Yes, in past two years

2. Yes, but it was more than two years ago

3. No 🡪 **GO TO QUESTION M13**

M12. How many times has your spouse or significant other been deployed for more than 30 days in a row? _____

M13. Was your spouse or significant other ever deployed to a combat zone?

1. No🡪 **GO TO QUESTION M15**

2. Yes, deployed to Iraq and/or Afghanistan

3. Yes, deployed to a combat zone other than Iraq or Afghanistan

M14. During their active duty career, how many times was your spouse or significant other deployed to a combat zone?

____________

M15. Is your spouse or significant other currently deployed?

1. Yes

2. No **🡪 GO TO QUESTION DS1**

M16. In what month and year did your spouse or significant other leave for his/her most recent deployment?

|  |  |  |
| --- | --- | --- |
| Month |  | Year |

M17. When do you expect your spouse or significant other to return?

1. Within the next 3 months

2. In 4–6 months

3. In 7–9 months

4. In 10–12 months

5. More than 12 months from now

The last set of questions asks about your use of dietary supplements.

DS1. During the past 7 days, have you taken a multi-vitamin or multi-mineral supplement? These products are things like One-A-Day, Mega-Vitamin, Centrum A-To-Zinc, and other products that contain a variety of different vitamins, minerals, and may also contain other things as well.

1. Yes

2. No **🡪 GO TO QUESTION DS4**

DS2. If yes, what kinds are you taking (describe and provide brand name(s) if known) ________________________________________________________________________

DS3. How often do you usually take the supplements (describe, such as 1 pill daily or every other day)?

________________________________________________________________________

DS4. During the past 7 days, have you taken a specialized or single-ingredient vitamin or mineral supplement such as Vitamin C, Vitamin E, iron, or calcium?

1. Yes

2. No**🡪 END SURVEY**

DS5. If yes, what kinds are you taking (describe and provide brand name(s) if known) ________________________________________________________________________

DS6. How often do you usually take the supplements (describe, such as 1 pill daily or every other day)?

________________________________________________________________________

**See master log to schedule a follow-up interview.**

-------------------------------------------------

For study personnel use only:

Weight: __ __ __ . __ lbs

Height: __ __ . __ inches
